# Can we reduce bias in open-label trials when blinded outcome assessment is not possible? An example from the trigger trial

**DOI:** 10.1186/1745-6215-16-S2-O71

**Published:** 2015-11-16

**Authors:** Brennan Kahan, Vipul Jairath

**Affiliations:** 1 Pragmatic Clinical Trials Unit, Queen Mary University of London, London, UK; 2 Translational Gastroenterology Unit, Nuffield Department of Medicine, University of Oxford, Oxford, UK

## 

Blinded outcome assessment is a key component of randomised trials, as unblinded assessment can lead to bias. However, in some circumstances blinded assessment may be difficult to achieve. In these situations, it may be useful to modify the outcome definition to remove the most subjective elements, thereby reducing the risk of bias.

This is the approach used in TRIGGER, an open-label cluster-randomised trial in patients with acute upper gastrointestinal bleeding. The primary clinical outcome was further bleeding. Blinded outcome assessment was impossible, as all clinicians throughout a hospital were aware of the treatment allocation due to the use of cluster-randomisation, and given the emergency nature of the condition, it was not possible to compile relevant information to send to an adjudication committee in a blinded matter. We therefore modified the outcome definition to remove subjective events (e.g. if a patient vomited blood, whether it was ‘fresh’ enough to indicate a new bleed), leaving only relatively objective events (the presence vs. absence of blood in the patient's upper gastrointestinal tract, based on a visual inspection by endoscopy).

We collected both outcomes (including vs. removing subjective events) during the trial, and compared the estimated treatment effects from both. Including subjective events led to an odds ratio (OR) of 0.83 (95% CI 0.50 to 1.37), compared to an OR of 0.50 (95% CI 0.32 to 0.78) after removing subjective events. The ratio of odds ratios was 1.66, indicating that including subjective events may biased the treatment effect upwards by 66%.

